# Editorial: Gut microbiota modulation by dietary fiber on human health: Processes and mechanisms

**DOI:** 10.3389/fmicb.2023.1160746

**Published:** 2023-02-23

**Authors:** Xiaowei Zhang, Tao Feng, Yunus Emre Tuncil

**Affiliations:** ^1^School of Health Science and Engineering, University of Shanghai for Science and Technology, Shanghai, China; ^2^School of Perfume and Aroma Technology, Shanghai Institute of Technology, Shanghai, China; ^3^Department of Food Engineering, Necmettin Erbakan University, Konya, Türkiye

**Keywords:** gut microbiota, dietary fiber, bioactive compounds, anti-obesity, short-chain fatty acids (SCFA), structural feature, gut health

The human gut is colonized by trillions of microorganisms that play a crucial role in maintaining the health (Shahi et al., 2017). The microbiome contributes to various physiological processes, such as digestion and absorption of nutrients, regulation of the immune system, and protection against harmful pathogens (Kho and Lal, 2018). An imbalance in the gut microbiome, known as dysbiosis, has been linked to a range of health problems, including inflammatory bowel disease, obesity, type 2 diabetes, and even certain neurological disorders (Sun and Chang, 2014). Maintaining a healthy gut microbiome through a balanced diet, probiotics, and prebiotics can promote overall health and prevent the development of various health issues. Dietary fiber (DF), which serves as a source of food for gut microbiota, is one of the most important factors in modulating gut microbiota composition (Zhang et al., 2021). The gut bacteria could ferment DFs to produce short-chain fatty acids (SCFAs) that can be used as an energy source and as a signaling molecule that helps regulate various aspects of gut health, including maintaining gut integrity, modulating the immune system, and reducing inflammation (Koh et al., 2016).

In this Research Topic, recent findings in the modulation of gut microbiota through DFs and bioactive compounds and their potential to promote gut health are pointed out. It comprised five papers, the role of two polysaccharides (He et al.; Zhang et al.), one flavonoid (Meng et al.), DF and phenolic acid (Li et al.), and SCFAs (Zhan et al.) in maintaining gut health and preventing various health issues were explored.

The study conducted by Zhang et al. aimed to examine the impact of *Lyophyllum decastes* (Fr.) Singer polysaccharides (LDSPs) on gut microbiota and their metabolites in the *in vitro* human fecal fermentation. LDSPs produced a significantly higher level of total SCFAs, particularly propionate and butyrate, compared to inulin, the positive control. The microbiota analysis revealed a distinct shift in the gut microbial composition between the LDSPs group and the inulin group. Notably, the LDSPs treatment increased the relative abundance of butyrogenic bacteria such as *Blautia, Roseburia*, and *Faecalibacterium*, and a positive correlation was found between butyrate production and these bacteria. The results suggested that LDSPs have the potential to demonstrate health benefits as a prebiotic.

He et al. investigated the anti-obesity activity of a polysaccharide (TP) extract from *Tremella fuciformis*, a medicinal and edible fungus. TP supplementation could significantly reduce weight gain, fat accumulation, blood glucose, hyperlipidemia, and inflammation induced by the high-fat diet (HFD) in mice. This was achieved by regulating gut microbiota disturbance, improving gut barrier integrity, and increasing the production of SCFAs. The *Firmicutes*/*Bacteroidetes* ratio was reduced while the relative abundances of *Muribaculaceae, Oscillospiraceae, Prevotellaceae*, and *Bacteroidaceae* were increased with TP treatment. The anti-obesity effect of TP was also transferable through fecal microbiota transplantation, further confirming that the anti-obesity activity of TP was achieved *via* modulation of gut microbiota. Overall, the study suggests that TP is a promising prebiotic for the prevention and treatment of metabolic diseases such as hyperglycemia and obesity by targeting the gut microbiota.

Besides DF, bioactive compounds such as polyphenols could also modulate gut microbiota and provide health benefits (Ozdal et al., 2016). In the study of Meng et al., eriocitrin, a flavonoid present in lemon fruit, was applied to investigate its intestinal metabolic profile and effect on gut microbiota in mice. Eriocitrin and its six metabolites, including eriodictyol, homoeriodictyol, hesperetin, eriodictyol-3′-O-glucoside, hesperetin-7-O-glucoside, and eriodictyol-7-O-(6″-O-galloyl) glucoside, were detected in the colon contents. The level of all SCFAs, particularly butyrate, valerate, and hexanoate, were significantly increased in the eriocitrin intervention diet group. This, in turn, was accompanied by an increase in the abundance of butyrogenic bacteria such as *Lachnospiraceae*_UCG-006. Moreover, Spearman's rank correlation analysis showed that *Monoglobus, Faecalibacterium, Candidatus_Arthromitus, Lachnospiraceae*_UCG-006, *Gardnerella*, and *Lactobacillaceae*_HT002 were positively correlated with the most of the eriocitrin metabolites, while *Parasutterella* and *Muribaculaceae*_unclassified exhibited negative correlations. These findings suggest that eriocitrin has the potential to positively impact gut health through the modulation of gut microbiota and metabolism.

Furthermore, polyphenols were frequently either free or esterified bonded with DFs in foods, thus it is important to evaluate the mutual effect of polyphenols and DFs on the modulation of gut microbiota. In the study of Li et al., the impact of the carbohydrate structure and feruloylation of arabinoxylan (AX) on fermentability and capability to modulate gut microbiota was determined *in vitro*. Rice bran arabinoxylan (RAX), corn bran arabinoxylan (CAX), and their deferulyolated counterpart dRAX and dCAX, were subjected to an *in vitro* fecal fermentation model. The carbohydrate backbone and branches of AX played a more significant role in the fermentation pattern compared to feruloylation. Different *Bacteriodes* species were promoted by RAX and CAX, and the effect of feruloylation on butyrate production and alpha diversity varied depending on the carbohydrate structure. For instance, dRAX showed lower butyrate production, compared to RAX, while dCAX showed a higher butyrate production compared to CAX. This study highlights the importance of considering the combination of polyphenols and DFs in the modulation of gut microbiota.

SCFAs are the major metabolites of DFs in the colon and play a crucial role in the regulation of gut function (Koh et al., 2016). In the study of Zhan et al., the potential role of SCFAs in controlling enteric pathogens is summarized. By extensively promoting the acylation of key bacterial proteins and regulating the gut barrier function and immune status, SCFAs could prevent the invasion of pathogens. It was noted that the types, composition ratio, and concentration of SCFAs greatly affected their regulatory role in the gut. The need for further studies to fully understand the role of SCFAs in controlling enteric pathogens is highlighted. The results indicated that increasing the production of SCFAs through dietary interventions or supplementation may be a promising approach for controlling enteric pathogens and improving gut health.

Overall, these papers provide important insights into the effects of various dietary components on gut health and highlight the potential of polysaccharides, flavonoids, and SCFAs as prebiotics. However, further research is needed to fully understand the mechanisms behind these effects and to determine the optimal composition and dosage of these compounds for optimal gut health. Additionally, future studies should also consider the role of individual differences, such as genetics and diet, in shaping the gut microbiome and its response to dietary interventions. Taken together, the papers contribute to the growing body of evidence supporting the importance of gut health and the need for further research into the gut microbiome and its role in health and disease.

## Author contributions

XZ drafted and wrote the manuscript. TF and YT reviewed and revised the manuscript. All authors contributed to the manuscript and approved the submitted version.
